# Single-cell RNA-seq analysis identifies meniscus progenitors and reveals the progression of meniscus degeneration

**DOI:** 10.1136/annrheumdis-2019-215926

**Published:** 2019-12-23

**Authors:** Hao Sun, Xingzhao Wen, Hongyi Li, Peihui Wu, Minghui Gu, Xiaoyi Zhao, Ziji Zhang, Shu Hu, Guping Mao, Ruofan Ma, Weiming Liao, Zhiqi Zhang

**Affiliations:** 1 Department of Orthopedics, Sun Yat-Sen Memorial Hospital, Guangzhou, China; 2 Department of Joint Surgery, Sun Yat-sen University First Affiliated Hospital, Guangzhou, China

**Keywords:** cytokines, arthritis, fibroblasts

## Abstract

**Objectives:**

The heterogeneity of meniscus cells and the mechanism of meniscus degeneration is not well understood. Here, single-cell RNA sequencing (scRNA-seq) was used to identify various meniscus cell subsets and investigate the mechanism of meniscus degeneration.

**Methods:**

scRNA-seq was used to identify cell subsets and their gene signatures in healthy human and degenerated meniscus cells to determine their differentiation relationships and characterise the diversity within specific cell types. Colony-forming, multi-differentiation assays and a mice meniscus injury model were used to identify meniscus progenitor cells. We investigated the role of degenerated meniscus progenitor (DegP) cell clusters during meniscus degeneration using computational analysis and experimental verification.

**Results:**

We identified seven clusters in healthy human meniscus, including five empirically defined populations and two novel populations. Pseudotime analysis showed endothelial cells and fibrochondrocyte progenitors (FCP) existed at the pseudospace trajectory start. Melanoma cell adhesion molecule ((MCAM)/CD146) was highly expressed in two clusters. CD146+ meniscus cells differentiated into osteoblasts and adipocytes and formed colonies. We identified changes in the proportions of degenerated meniscus cell clusters and found a cluster specific to degenerative meniscus with progenitor cell characteristics. The reconstruction of four progenitor cell clusters indicated that FCP differentiation into DegP was an aberrant process. Interleukin 1β stimulation in healthy human meniscus cells increased CD318+ cells, while TGFβ1 attenuated the increase in CD318+ cells in degenerated meniscus cells.

**Conclusions:**

The identification of meniscus progenitor cells provided new insights into cell-based meniscus tissue engineering, demonstrating a novel mechanism of meniscus degeneration, which contributes to the development of a novel therapeutic strategy.

**Key messages**

**What is already known about this subject?**

- The cell types of meniscus contain chondrocyte-like morphology cells and fibroblast-like cells. However, the variety of cell types and corresponding biological markers, as well as the biological targets for the treatment for meniscus degeneration remain elusive.

**What does this study add?**

- This study provides comprehensive census of human meniscus cells using single-cell RNA sequencing, and demonstrating CD146+ meniscus cells are stem/progenitor cells.
- Interleukin 1β induced activation of degenerated meniscus progenitor cells (DegP) is a potential mechanism contributing to meniscus degeneration.

**How might this impact on clinical practice or future developments?**

- CD146+ meniscus cells have potential in meniscus tissue engineering, and DegP could be a possible therapeutic target for meniscus degeneration.

## Introduction

The menisci of mammals are crescent‐shaped tissues, comprised a medial and a lateral component.^1^ The meniscus plays an important role in joint stability, shock absorption, distribution of contact forces, joint lubrication and proprioception. The vascularisation of the meniscus decreased with ageing. The meniscus is fully vascularised during prenatal development and shortly after birth, however, only 10%–25% of mature meniscus contains blood vessels.^2^ According to these differences in blood supply, the meniscus can be distinguished by the outer vascular region (red zone), inner avascular region (white zone) and the red-white zone between the red and white zones. The outer zone of the meniscus contains 90% type I collagen while the inner zone contains 60% type II collagen and 40% type I collagen.^3^

The cell types of the meniscus are heterogeneous, wherein the inner region contains chondrocyte-like morphology cells and the outer region fibroblast-like cells.^4^ Recently, stem/progenitor cells were suggested to be present in the meniscus to promote meniscus injury repair.^5 6^ Gamer *et al* isolated meniscus stem/progenitor cells by meniscus explant culture in vitro, characterising these cells with clonogenicity properties and abundantly expressed CD44 and Sca-1.^7^ However, the cell-type composition and cell distribution in the menisci, as well as biochemical markers for meniscus stem cell/progenitor for use in tissue engineering, remain to be elucidated.

The relationship between meniscus degeneration and knee osteoarthritis (OA) is complex. Meniscus degenerative tears were found to be associated with increased cartilage loss in the same compartment, especially in posterior horn tears.^8 9^ Fuller *et al* found that both inner and outer zone meniscal cells are responsive to the inflammatory cytokines IL-1α and TNF-α in an ovine *in* *vitro* model, which leads to cytokine-induced collagenolysis and aggrecanolysis.^10 11^ These studies demonstrated the importance of meniscus degeneration in OA development and its contribution to joint disease in general. Degenerative meniscus accompanied by water content increased the wet weight, while collagen and total glycosaminoglycans (GAG) decreased.^12^ However, the variety of cell types and corresponding biological markers, as well as the biological targets for the treatment for meniscus degeneration, have not yet been fully determined.

Single-cell RNA sequencing (scRNA-seq) is a well-established and powerful method to investigate transcriptomic cell-to-cell variation, which can be used to identify various cell types and provide insights into physiological and pathological processes.^13 14^ Here, we used scRNA-seq to chart a comprehensive census of meniscus cells. We identified various cell subsets and their gene signatures to determine their differentiation relationships and characterise diversity within specific cell types. We also demonstrated the existence of meniscus stem/progenitor cells and their corresponding marker genes. Finally, we investigated the integral influence of meniscus degeneration on meniscus cellular heterogeneity and identified a potential therapeutic target.

## Materials and methods

### Isolation of human meniscus cells

Human meniscus tissues were dissected away from the synovium, and then cut into small pieces. Next, these small pieces were digested by 4 mg/mL protease (Roche 11459643001) for 1 hour and 2 mg/mL collagenase P (Roche 11213873001) for 6–10 hours.

## Results

### scRNA-seq census of healthy human meniscus identified seven distinct cell populations

**Figure 1:**
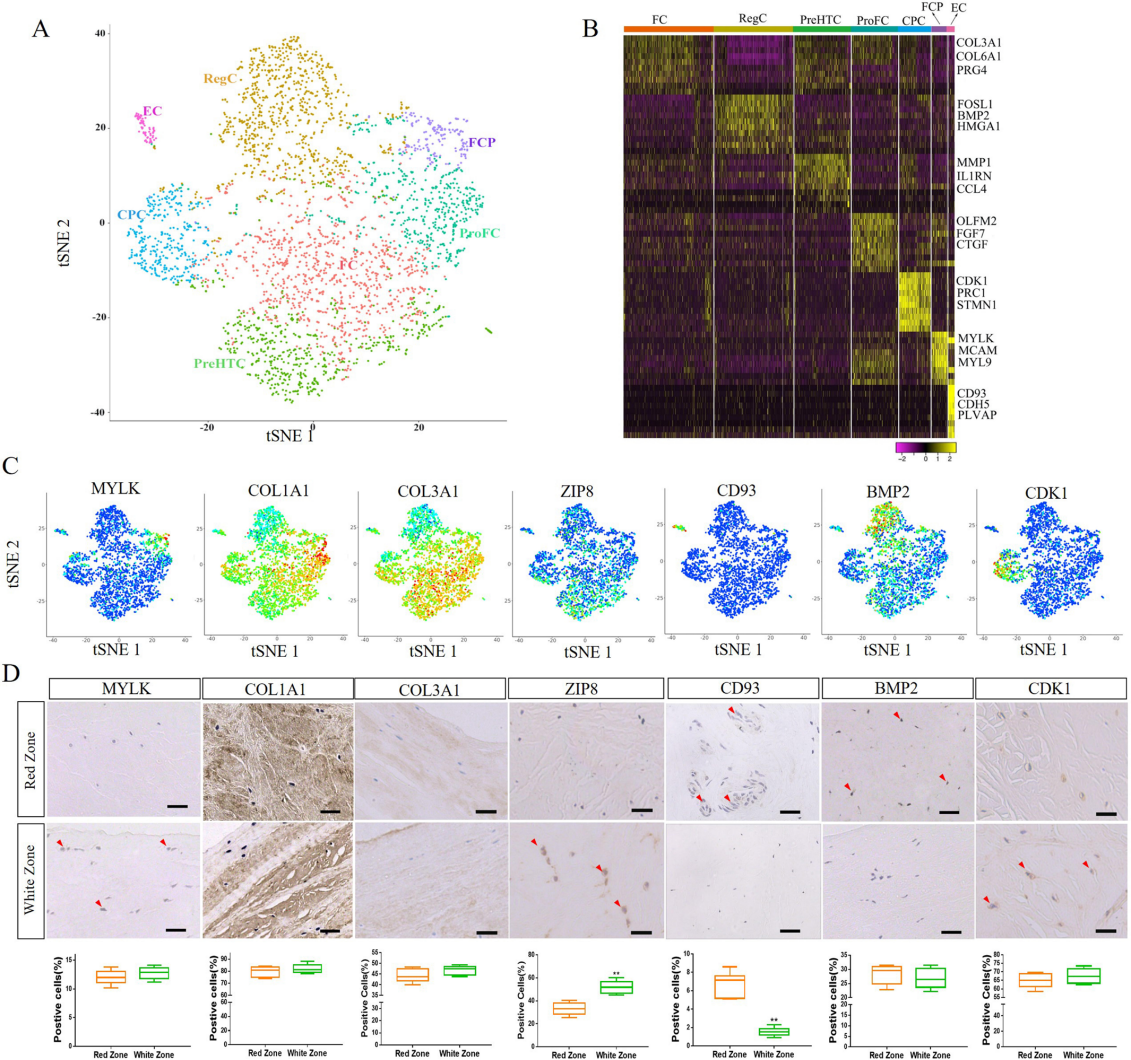
A single-cell atlas of healthy human meniscus. (A) Seven healthy human meniscus cell clusters. t-Distributed stochastic neighbour embedding (t-SNE) of 3639 cells (mixed with cell fractions, n=3), annotated post-hoc and coloured by clustering. (B) Heatmap revealing the scaled expression of differentially expressed genes for each cluster. (C) Dot plots showing the expression of the indicated markers for each cell cluster on the t-SNE map. (D) Representative immunohistochemistry staining of MYLK, COL1A1, COL3A1, ZIP8, CD93, BMP2 and CDK1 in white and red zones of healthy human meniscus tissues, and quantification of positive cells displayed by box plot (n=6). Scale bar, 50 µm. **p<0.01. CPC, cartilage progenitor cells; EC, endothelial cells; FC, fibrochondrocytes; FCP, fibrochondrocyte progenitors; PreHTC, prehypertrophic chondrocytes; ProFC, proliferate fibrochondrocytes; RegC, regulatory chondrocytes.

Our results showed that the cell quality was satisfactory for our single cell sequencing (online supplementary figure S1). To determine the cellular composition of human meniscus cells, we profiled meniscus cells from healthy human meniscus (n=3) using scRNA-seq. Unbiased clustering of the meniscus resulted in seven clusters originating from healthy human meniscus, including five empirically defined populations and two novel populations (figure 1A). Concretely, the following cells were identified: (1) endothelial cells (EC, expressing CD93 and CDH5),^15^ (2) cartilage progenitor cells (CPC, expressing CDK1 and BIRC5),^16^ (3) regulatory chondrocytes (RegC, expressing BMP2 and FOSL1),^17^ (4) fibrochondrocytes (FC, expressing COL1A1, COL3A1 and COL6A1),^16 18^ (5) prehypertrophic chondrocytes (PreHTC, expressing MMP1 and TNFAIP6),^19 20^ (6) fibrochondrocyte progenitors (FCP, expressing both the fibrochondrocyte genes COL1A1 and COL3A1 and the mesenchymal stem cell marker genes MCAM and MYLK)^21^ and (7) proliferate fibrochondrocytes (ProFC, expressing both the fibrochondrocyte gene COL1A1 and growth factors FGF7 and CTGF)^22^ (figure 1B, C). FC and RegC were abundant, while FCP and EC were relatively rare.

10.1136/annrheumdis-2019-215926.supp1 Supplementary data

To study the distribution of different cell clusters, we used immunohistochemistry to detect marker gene expression. MYLK, a marker gene of FCP, was mainly expressed on the meniscus surface, while the RegC gene marker BMP2 was mainly expressed in the middle of the meniscus. CD93 is the marker gene of EC and is mainly expressed around the vessels in the red zone, while the PreHTC marker ZIP8 was mainly expressed in white zone. No difference was found between the red and white areas regarding the expression of ProFC marker COL1A1, FC marker COL3A1 and CPC marker CDK1 (figure 1D).

### Identification of population of human meniscus progenitor cells

To investigate the relationship between the different cell clusters, we used the Monocle method to reconstruct the pseudospace trajectory. We found that EC and FCP existed at the start of the pseudospace trajectory, and ProFC located in front of FC, while PreHTC was behind of FC. FC and CPC were distributed along the trajectory, and RegC was mainly distributed at the end (online supplementary figure S2A, B).

10.1136/annrheumdis-2019-215926.supp2 Supplementary data

**Figure 2:**
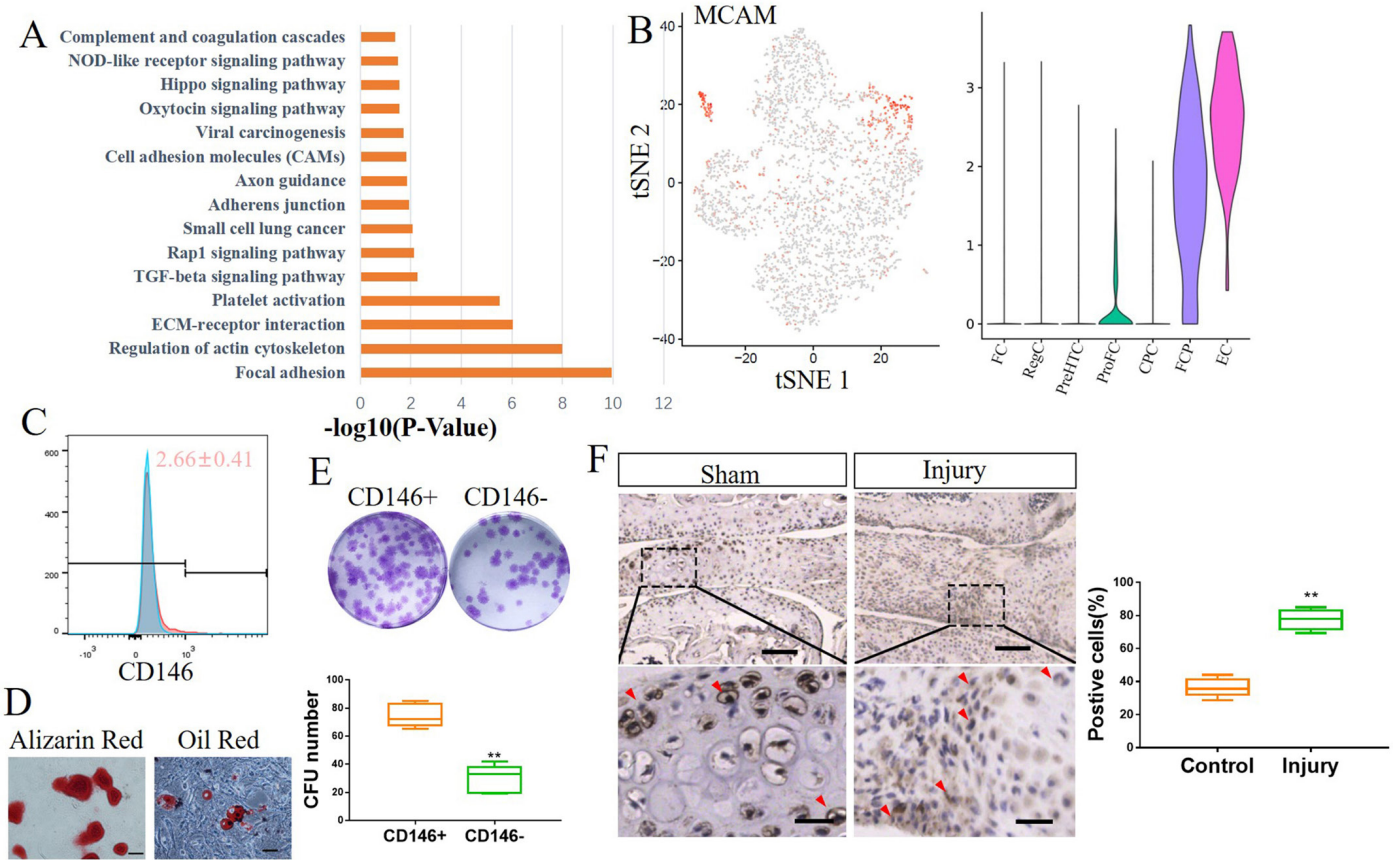
Identification of human meniscus progenitor cells. (A) The 15 most upregulated signal pathways in FCP. (B) Dot plots showing the MCAM expression on t-distributed stochastic neighbour embedding (t-SNE) map and Vin plot. (C) CD146 expression in healthy human meniscus cells determined by flow cytometry (mean±SD; n=3). (D) Alizarin red staining and oil red staining for CD146+ meniscus cells induced to osteogenic differentiation or adipogenic differentiation, respectively (n=5). Scale bar, 50 µm. (E) Colony-forming analysis of CD146+ and CD146− healthy human meniscus cells and quantification. n=5, **p<0.01. (F) IHC staining of MYLK in mice meniscus injury model, and quantification of positive cells. Scale bars, 200 µm (top) and 50 µm (bottom). n≥6, **p<0.01. CFU colony forming unit; CPC, cartilage progenitor cells; EC, endothelial cells; FC, fibrochondrocytes; FCP, fibrochondrocyte progenitors; NOD, nucleotide-binding oligomerisation domain; PreHTC, prehypertrophic chondrocytes; ProFC, proliferate fibrochondrocytes; RegC, regulatory chondrocytes.

Since FCP existed at the start of the pseudospace trajectory, we investigated whether it had properties characteristic of a progenitor. Pathway analysis showed that pathways involved in focal adhesion, extracellular matrix (ECM)–receptor interaction and TGFβ signalling were activated (figure 2A). FCP expressed the mesenchymal stem cell marker MCAM (CD146) (figure 2B), as well as classical markers of myofibroblasts, including ACTA2, MYLK and MYL9 (online supplementary figure S2C).

We isolated the CD146+ primary human meniscus cells using fluorescence-activated cell sorting (FACS) and found that the proportion of CD146+ meniscus cells was near 2.7% (figure 2C). CD146+ cells had the ability to differentiate into various cell lineages, including osteoblasts and adipocytes (figure 2D). Next, we examined the clonogenicity of CD146+ cells. A total of 2000 CD146+ and CD146– cells were seeded in 12-well plates and cultured for 7 days. After culturing, the number of colonies in the CD146+ group was significantly higher than that of the colonies in the CD146– cells group (figure 2E). The fact that the cells in the CD146– group were able to form colonies suggests that another cell cluster may also have progenitor properties. CD93 is the specific marker of ECs, so we used FACS to obtain CD146+/CD93+ meniscus cells (EC) and CD146+/CD93– meniscus cells (FCP). Further experiments showed that these two clusters have progenitor properties (online supplementary figure S2D).

### Single-cell trajectory branch points correspond to FCP differentiation

**Figure 3:**
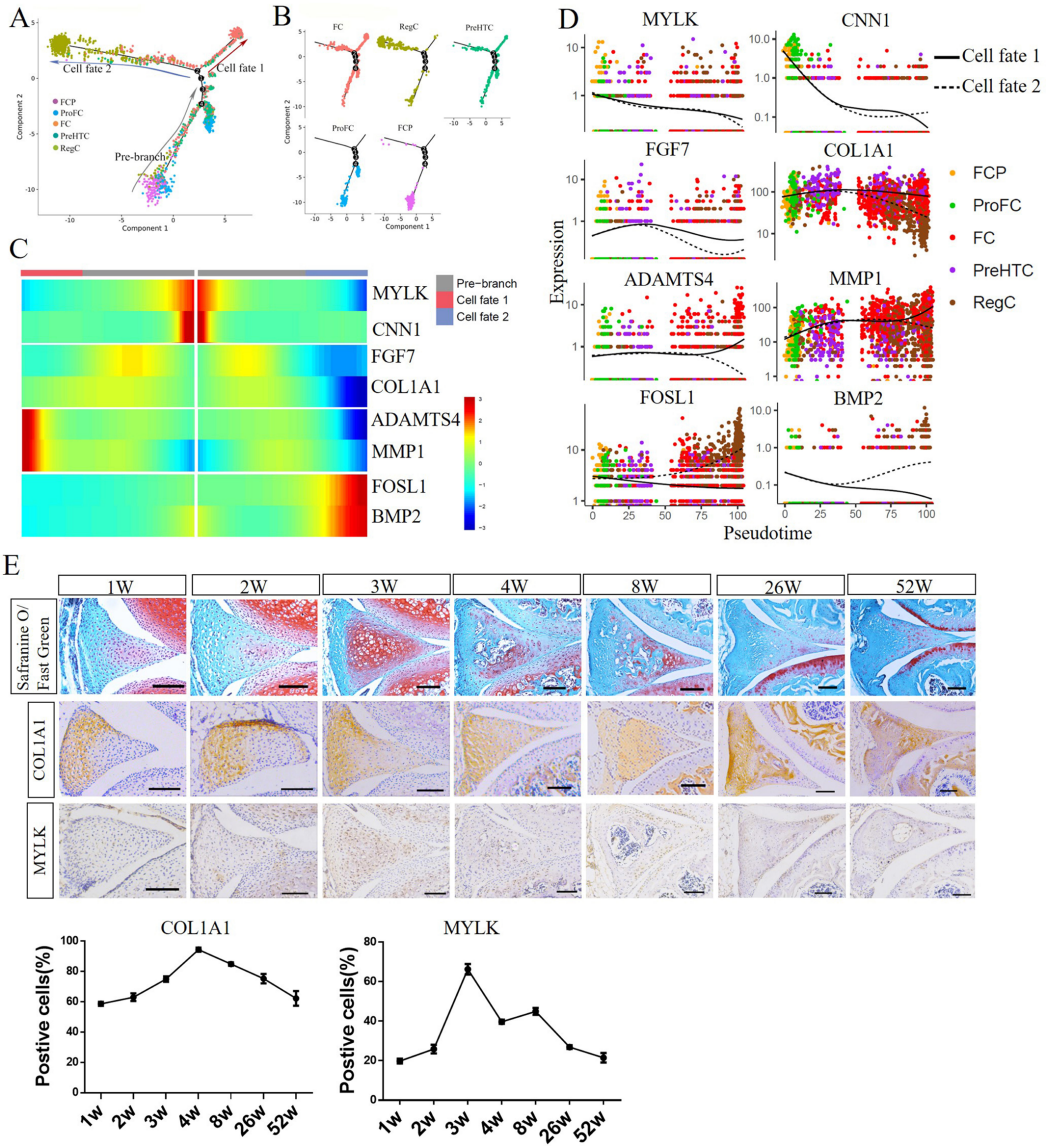
Single-cell trajectory branch points demonstrating FCP differentiation. (A, B) Monocle pseudotime trajectory showing the progression of FCP, ProFC, FC, PreHTC and RegC. (C) The expression of the genes in a branch-dependent manner. Each row indicates the standardised kinetic curves of a gene. The centre of the heatmap shows the kinetic curve value at the root of the trajectory. From the centre to the left of the heatmap, the kinetic curve progresses from the root along the trajectory to fate 1. Starting from the right, the curve from the root to fate 2. (D) Pseudotime kinetics of indicated genes from the root of the trajectory to fate 1 (solid line) and the cells up to fate 2 (dashed line). (E) Safranine O/Fast Green staining and immunohistochemistry staining of COL1A1 and MYLK in mice anterior meniscus at 1, 2, 3, 4, 8, 26 and 52 weeks, and quantification of positive cells (n≥3). Scale bar, 100 µm. CPC, cartilage progenitor cells; EC, endothelial cells; FC, fibrochondrocytes; FCP, fibrochondrocyte progenitors; PreHTC, prehypertrophic chondrocytes; ProFC, proliferate fibrochondrocytes; RegC, regulatory chondrocytes.

To study the differentiation of FCP into subset clusters and the corresponding gene expression, we selected FCP, ProFC, FC, PreHTC and RegC to construct a new trajectory containing two termini corresponding to two distinct cell fates (figure 3A). The root of the trajectory was mainly populated by FCP and ProFC, while the two termini of the tree were populated by FC and PreHTC for fate 1, RegC and PreHTC for fate 2 (figure 3B). Next, we assessed the expression of genes regulated during FCP differentiation in cells at fates 1 and 2 of the trajectory. The expression of MYLK, CNN1, FGF7 and COL1A1 were found to be similar. While MYLK and CNN1 expression was markedly reduced from the root through to both fates, FGF7 and COL1A1 expression was upregulated early in FCP differentiation and downregulated in cells differentiating into both fates (figure 3B, C). ADAMTS4 and MMP1 were slightly upregulated at early stage differentiation, and notably upregulated in cells at fate 1 and downregulated at fate 2. On the contrary, FOSL1 and BMP2 expression slightly decreased at cells from the root to fate 1, but markedly increased in cells differentiating via fate 2.

To confirm the single-cell trajectory, we analysed the meniscus developmental process in vivo and studied the expression of marker genes in the mice meniscus at 1, 2, 3, 4, 8, 26 and 52 weeks. COL1A1 expression increased gradually after birth and peaked at 4 weeks, then decreased gradually with increasing age (figure 3E). MYLK expression decreased significantly with increasing age after 3 weeks (figure 3E). These expression patterns were consistent with two different fates of the trajectory, indicating that our scRNA-seq analysis correlated with the meniscus developmental process.

### Systemic comparison of the single cell landscape between healthy human meniscus and degenerated meniscus

**Figure 4:**
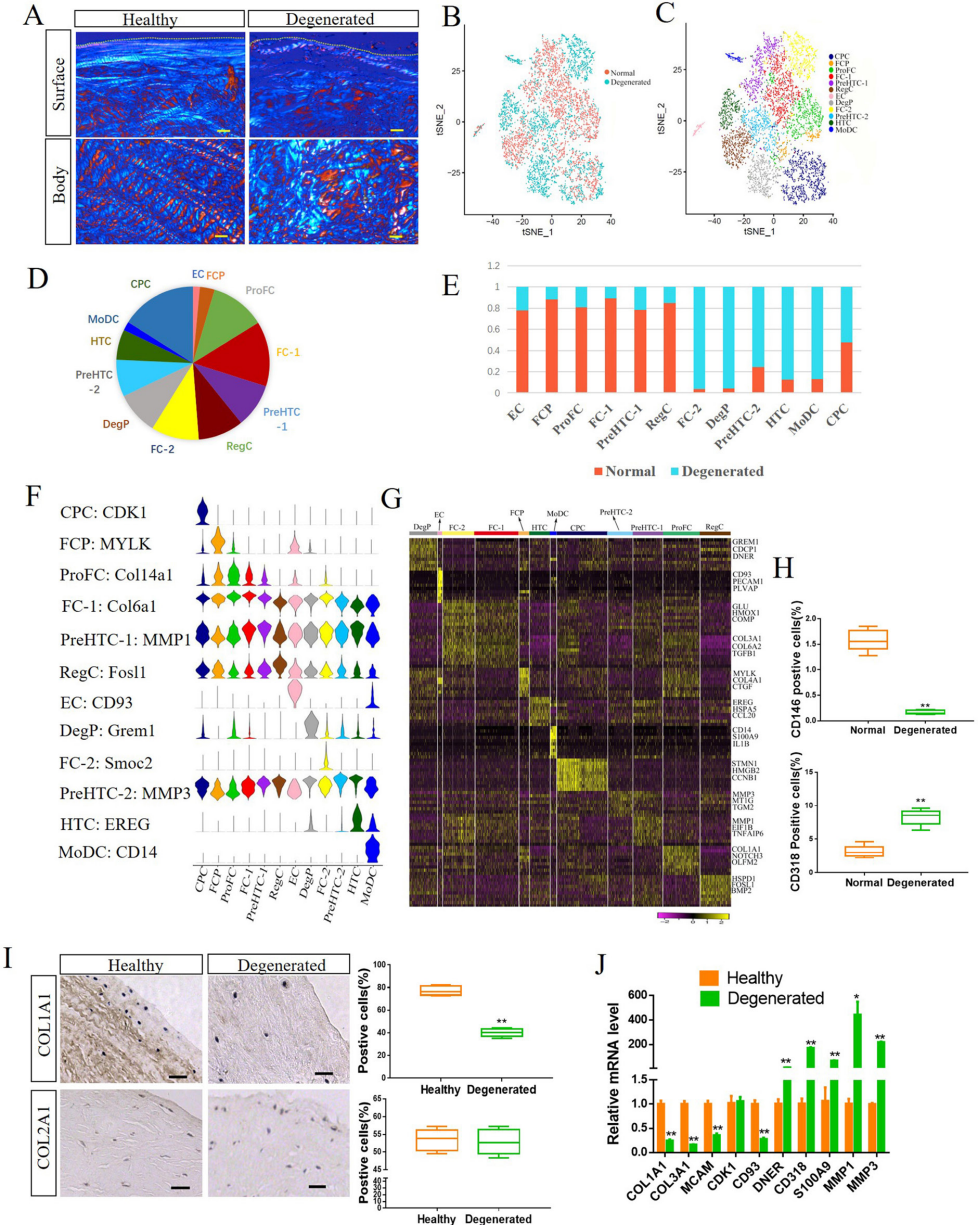
Comparison of the single cell landscape between healthy human meniscus and degenerated meniscus. (A) Representative polarised light microscopy images of healthy human and degenerated meniscus. The white and red colours in the angle images are 90° apart in orientation. Dashed lines indicate the surface of the meniscus. Scale bar, 100 µm. (B) Merged t-distributed stochastic neighbour embedding (t-SNE) of single-cell RNA sequencing of healthy meniscus cells and degenerated meniscus cells. (C) Twelve healthy human and degenerated meniscus cell clusters at t-SNE. (D) Proportion of each cluster to the total cells. (E) Proportion of healthy and degenerated meniscus cells in each cluster. (F) Expression of representative marker genes in Vin plot. (G) Heatmap revealing the scaled expression of differentially expressed genes for each cluster. (H) CD146 and CD318 expression in healthy human meniscus cells and degenerated meniscus cells determined by flow cytometry. n≥5, **p<0.01. (I) Representative IHC staining of COL1A1 and COL2A1 healthy human meniscus and degenerated meniscus, and quantification of positive cells. Scale bar, 50 µm. n≥5, **p<0.01. (J) The expression of indicated marker genes in human healthy meniscus cells and degenerated meniscus cells were detected by qRT-PCR. *p<0.05, **p<0.01, otherwise, not significant. n=3, *p<0.05, **p<0.01. CPC, cartilage progenitor cells; DegP, degenerated meniscus progenitor cells; EC, endothelial cells; FC, fibrochondrocytes; FCP, fibrochondrocyte progenitors; HTC, hypertrophic chondrocytes; MoDC, monocyte-derived dendritic cells; PreHTC, prehypertrophic chondrocytes; ProFC, proliferate fibrochondrocytes; RegC, regulatory chondrocytes.

To comprehensively assess the changes in the human meniscus during degeneration, we first evaluated the histological changes in degenerated meniscus. The healthy meniscus was negative for Safranine O staining, while the degenerative meniscus was positive for staining (online supplementary figure S3). In addition, the collagen fibre structure on the degenerated meniscus was disorganised (Figure 4A and online supplementary figure S4). Next, we compared the scRNA-seq between healthy meniscus and degenerated meniscus (figure 4B–E). As a result, we detected significant changes in the proportions of degenerated meniscus cell clusters, including three new clusters: (1) monocyte-derived dendritic cells (MoDC, expressing CD14 and S100A9),^23^ ^24^ (2) hypertrophic chondrocytes (HTC, expressing CCL20 and EREG)^25 26^ and (3) degenerated meniscus progenitor cells (DegP), which are found in degenerated meniscus and express skeletal stem cell marker, such as GREM1^27^ (figure 4C–G). Moreover, the proportion of EC and FCP expression was found to decrease in degenerated meniscus (figure 4E).

10.1136/annrheumdis-2019-215926.supp3 Supplementary data

10.1136/annrheumdis-2019-215926.supp4 Supplementary data

### Alignment of single-cell trajectories indicates DegP is a key element for meniscus degeneration

CDCP1 (CD318) is highly expressed in DegP (online supplementary figure S4A). As such, we isolated the CD318+ primary human degenerated meniscus cells by FACS to verify the progenitor capacity. CD318+ cells were found to form colonies and differentiate into various cell lineages (online supplementary figure S4B, C), wherein DegP was a special population with progenitor characteristics, and was mainly found in the degenerative meniscus.

**Figure 5:**
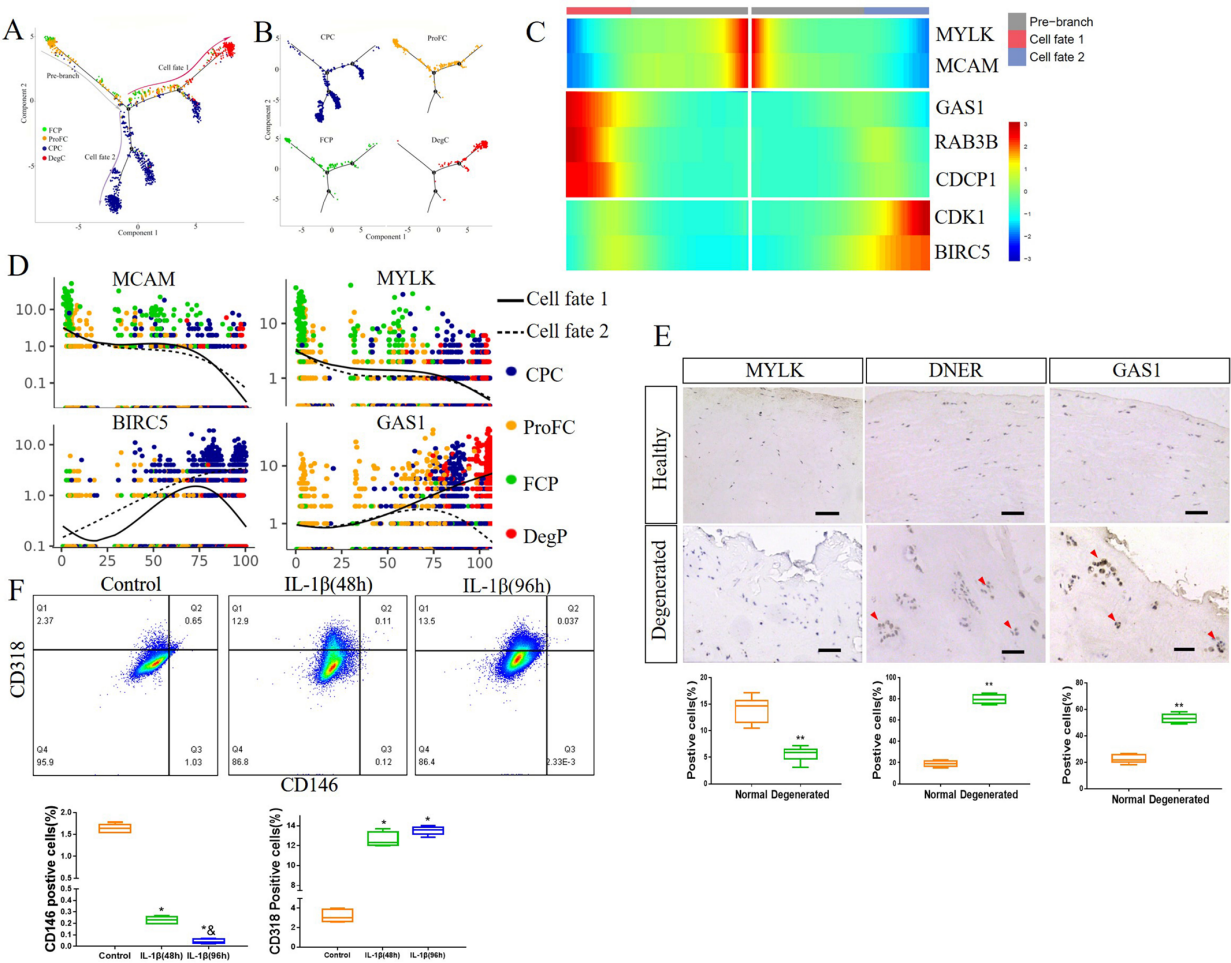
Identification of degenerated meniscus progenitor cells (DegP) as a key element for meniscus degeneration. (A, B) Monocle pseudotime trajectory showing the progression of FCP, ProFC, CPC and DegP. (C) From the centre to the left of the heatmap, the kinetic curve from the root along the trajectory to fate 1. Starting from the right, the curve from the root to fate 2. FCP markers MYLK and MCAM, DegP markers GAS1, Rab3B and CDCP1 and CPC markers CDK1 and BIRC5 expressed from the root to each branch. (D) Pseudotime kinetics of indicated genes from the root of the trajectory to fate 1 (solid line) and the cells up to fate 2 (dashed line). (E) Representative IHC staining of MYLK, GAS1 and DNER in healthy human meniscus and degenerated meniscus, and quantification of positive cells. Scale bar, 50 µm. n=6, **p<0.01. (F) Healthy human meniscus cells were treated with 5 ng/mL IL-1β for 48 hours or 96 hours. Phosphate buffer saline (PBS) was used as a negative control. CD146 and CD318 expression was determined by flow cytometry. n≥5, * versus control, p<0.05; & versus IL-1β (48 hours), p<0.05. CPC, cartilage progenitor cells; DeP, degenerated meniscus progenitor cell; FCP, fibrochondrocyte progenitors; ProFC, proliferate fibrochondrocytes.

Next, we selected four clusters with progenitor properties, including FCP, ProFC, CPC and DegP, to construct a new trajectory. The trajectory’s root was mainly populated by FCP and ProFC, while the two primary termini of the tree were populated by DegP and CPC for fate 1, and CPC for fate 2 (figure 5A, B). Although MCAM and MYLK were highly expressed at the root of the trajectory, their expression was markedly reduced along the root through to both fates 1 and 2 (figure 5C, D). BIRC5 and CDK1 were highly expressed at the end of fate 2, while GAS1, RAB3B and CDCP1 were highly expressed at the end of fate 1 (figure 5C, D and online supplementary figure S5A). However, in normal FCP differentiation, the expression of GAS1, RAB3B and CDCP1 was markedly reduced while progressing along from the root to both fates 1 and 2 (compared with figure 3, online supplementary figure S5B), indicating that fate 1 may be an aberrant cellular state during the degeneration process in meniscus.

10.1136/annrheumdis-2019-215926.supp5 Supplementary data

We also verified the expression of marker genes by IHC staining. MYLK, an FCP marker gene, was downregulated in degenerated meniscus, while the DegP marker genes GAS1 and DNER were upregulated in degenerated meniscus, especially in areas where meniscus lesions were accompanied by cell proliferation (figure 5E).

Proinflammatory mediators, such as IL-1β, appeared to directly influence the degradative processes in the meniscus.^28 29^ Therefore, we used IL-1β (5 ng/mL) to stimulate healthy human meniscus cells for 48 and 96 hours to detect any changes in CD146+ cells and CD318+ cells. IL-1β stimulation led to a significant reduction in CD146+ cells with an increasing stimulation time, while CD318+ cells significantly increased (figure 5F). We also used IL-1β to stimulate degenerated human meniscus cells and get similar results (online supplementary figure S5C). These results suggested that the decrease of CD146+ cells and the increase of CD318+ cells caused by various pathogenic factors such as IL-1β, may be an important mechanism of meniscus degeneration.

### Activation of TGFβ signalling pathway attenuates the increase in CD318+ cells in degenerated meniscus

**Figure 6:**
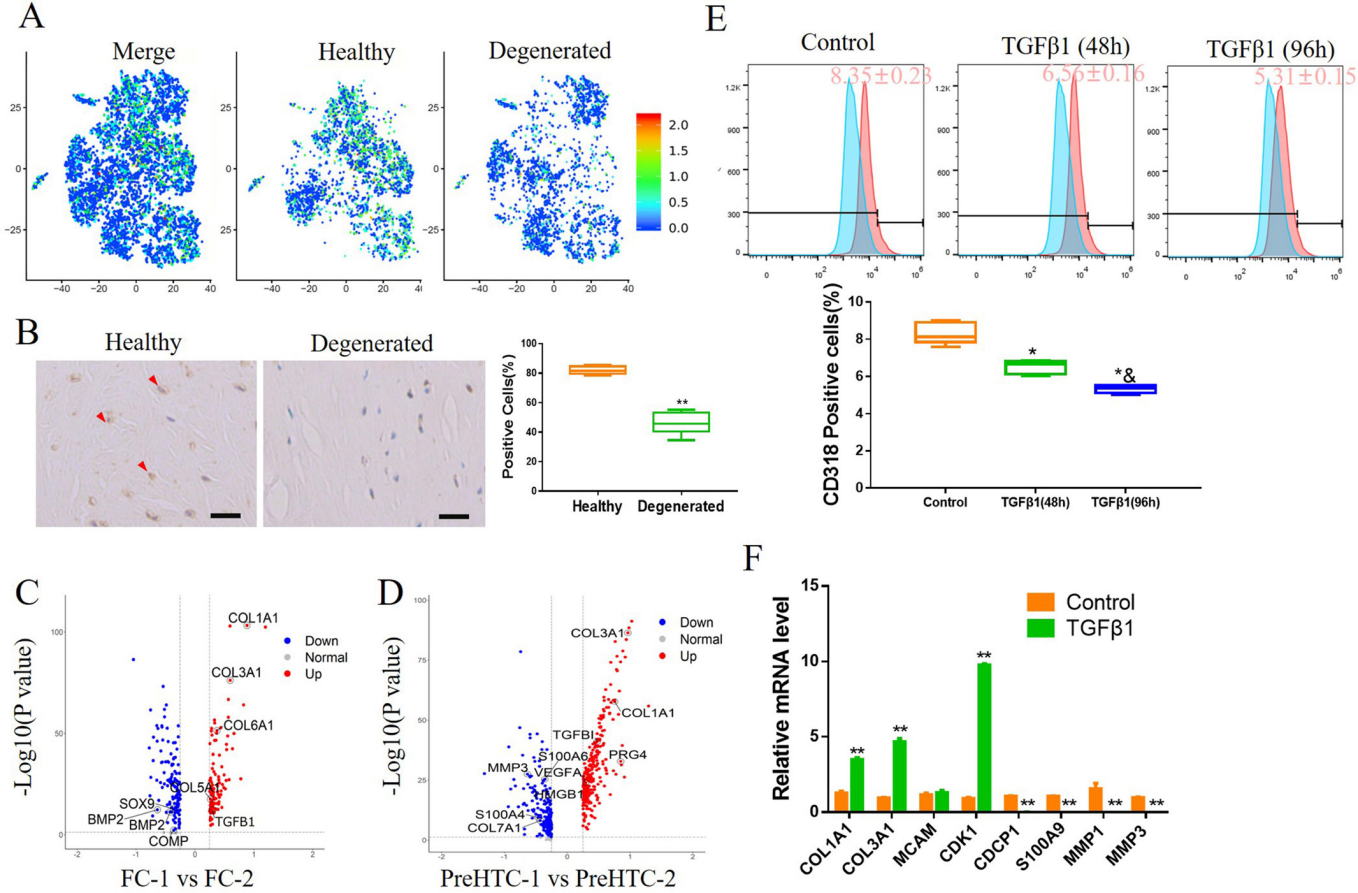
Activation of TGFβ signalling pathway attenuates the increase in CD318+ cells in degenerated meniscus. (A) The expression of TGFβ1 on merged and split t-distributed stochastic neighbourembedding map. (B) IHC staining of TGFβ1 on human healthy meniscus and degenerated meniscus. n=6, **p<0.01. (C) Volcano plot comparing the gene expression between FC-1 and FC-2. Each plot represents one gene. (D) Volcano plot comparing the gene expression between PreHTC-1 and PreHTC-2. Each plot represents one gene. (E) Human degenerated meniscus cells were treated with 5 ng/mL TGFβ1 for 48 hours or 96 hours. PBS was used as a negative control. CD318 expression was determined by flow cytometry (n≥5). * vs control, p<0.05; & vs TGFβ1 (48 hours), p<0.05. (F) Human degenerated meniscus cells were treated with 5 ng/mL TGFβ1 or PBS as negative control. The expression of indicated marker genes were detected by qRT-PCR. n=3, **p<0.01.

Previous studies have shown that the activation of TGFβ signalling enhances the differentiation ability of meniscus progenitors.^30 31^ Our scRNA-seq analysis and IHC staining showed that TGFβ1, a ligand of the transforming growth factor-β (TGFβ) signalling pathway, was highly expressed in healthy meniscus cells (figure 6A, B). We also compared the differences in gene expression between FC-1 and FC-2, PreHTC-1 and PreHTC-2, two cell types found in both healthy and degenerated meniscus. Compared with the clusters mainly found in degenerated meniscus (FC-2 and PreHTC-2), the clusters found in healthy meniscus (FC-1 and PreHTC-1) were upregulated by the TGFβ signalling pathway (online supplementary figure S6) and highly expressed COL1A1, COL3A1 and TGFβ1 (figure 6C, D). Next, we investigated the effect of TGFβ1 on DegP. Primary human degenerated meniscus cells were treated with 5 ng/mL TGFβ1 for 48 hours or 96 hours. Flow cytometry demonstrated that TGFβ1 treatment significantly reduced the number of CD318+ cells in a time depend manner (figure 6E), and qRT-PCR showed TGFβ1 treatment significantly increasing COL1A1, COL3A1 and CDK1 expression while decreasing CD318, S100A9, MMP1 and MMP3 expression (figure 6F), indicating that TGFβ1 may be able to delay the degeneration of meniscus.

10.1136/annrheumdis-2019-215926.supp6 Supplementary data

## Discussion

An increasing number of studies are supporting the idea that cell-based strategies effectively improve meniscus repair and regeneration.^32 33^ However, it is still not clear which cell type is most effective for meniscus repair.^34 35^ Recently, meniscus stem/progenitor cells have been considered as the most suitable cell type for meniscus injury repair due to them having the same tissue origin and histocompatibility,^36 37^ however, the characteristics, marker genes and isolation methods of human meniscus progenitor cells have not yet been fully elucidated. Gamer *et al* isolated meniscus progenitor cells from mice meniscus grown in explant cultures, and carried out flow cytometry analysis to show that these cells highly expressed CD44 and Sca-1.^7^ Shen *et al* digested human meniscus using collagenase and seeded the cells at a low density to form colonies. The subsequent flow cytometry analysis showed that these cell highly expressed CD90 (THY1) and CD105 (ENG), and the intra-articular injection of these cells promoted rat meniscus regeneration and ameliorated OA.^37^ Our scRNA-seq results also show the high expression of CD90 and CD105 in FCP, however, they were also highly expressed in FC-1 and FC-2. Thus, CD90 and CD105 were not markers specific to meniscus progenitor cells.

In our scRNA-seq results, EC was found to exist at the start of the pseudospace trajectory, which plays an important role in the development, degeneration and repair of the meniscus.^38 39^ Miller and Rydell isolated meniscus EC for the first time in 1993, and proved these cells had the ability to self-renew and maintain their characteristics after 10 passages.^40^ EC is not only able to generate vessels to maintain blood supply, but also promote the migration of meniscus cells. Yuan *et al* found that EC could enhance meniscus cell migration by activating endothelin signalling.^41^ Notably, we identified CD146 specifically expressed in EC and FCP, suggesting that CD146+ cells can be used in cell-based scaffolding for meniscus injury repair, and may also be a target for recruitment of meniscus progenitor cells by growth factors to participate in meniscus injury repair in cell-free strategies.

We identified three cell clusters specific to degenerated meniscus, including MoDC, HTC and DegP, where DegP is a novel cluster and has the characteristics of progenitor cells. Our scRNA-seq demonstrated that the expression of DegP markers, such as GAS1, RAB3B and CD318, increased rapidly at the end of the differentiation of FCP to DegP, which was contrary to the normal differentiation procedure, suggesting that this differentiation process was the result of an aberrant cellular state. IHC staining also showed that GAS1 and RAB3B were highly expressed in meniscus with severe lesions, which was accompanied by cell proliferation. CD318 has been previously demonstrated to be highly expressed in haematopoietic progenitors^42^ and muscle progenitors.^43^ Iwata *et al* revealed that CD318 is a CD146 negative subset of bone marrow fibroblasts and regulates cytokine expression.^44^ Previous studies have shown that inflammatory cytokines such as IL-1β and TNF-α induce meniscus metabolic responses and result in degeneration.^28 45^ In our study, IL-1β was used to induce the inflammatory response in human meniscus cells. We demonstrated that IL-1β decreased CD146+ cells and increased CD318+ cells in both healthy and degenerated meniscus cells. These results demonstrate that DegP plays a crucial role in meniscus degeneration and may be used as a marker to evaluate meniscus degeneration or as a target for the treatment of meniscus degeneration.

TGFβ is widely used in meniscus tissue engineering, owing to its promotion of meniscus injury repair and regeneration through the promotion of fibrochondrocyte proliferation and recruitment of meniscus progenitor cells.^30 46 47^ TGFβ also regulates the meniscus degeneration process, while the postnatal deletion of TGFβ signalling reporter ALK5 accelerates meniscus degeneration.^31^ Our scRNA-seq results showed that TGFβ1 is highly expressed in FC-1 and FC-2, and that its overall expression in degenerated meniscus is decreased. Treatment with TGFβ1 has been previously found to enhance the mechanical properties of tissue-engineered fibrocartilage.^48^ Our results revealed that TGFβ1 attenuated the proportion of CD318+ cells in human degenerated meniscus, suggesting that TGFβ1 may be used to suppress meniscus degeneration.

In conclusion, our scRNA-seq results provided a clearer and more consistent definition of the cellular components of human meniscus, and the ways in which specific clusters contribute to meniscus development and aberrant degeneration. Our analysis identified the meniscus progenitors with potential in meniscus tissue engineering. We also demonstrated an important mechanism of meniscus degeneration and provided experimental evidence for a therapeutic strategy.

10.1136/annrheumdis-2019-215926.supp7 Supplementary data

10.1136/annrheumdis-2019-215926.supp8 Supplementary data
