# Supplementary figures and images for: Single-cell RNA-seq analysis identifies meniscus progenitors and reveals the progression of meniscus degeneration
Source: Ann Rheum Dis. 2019 Dec 23;79(3):408–17. doi: 10.1136/annrheumdis-2019-215926 (PMC7034356; doi:10.1136/annrheumdis-2019-215926)

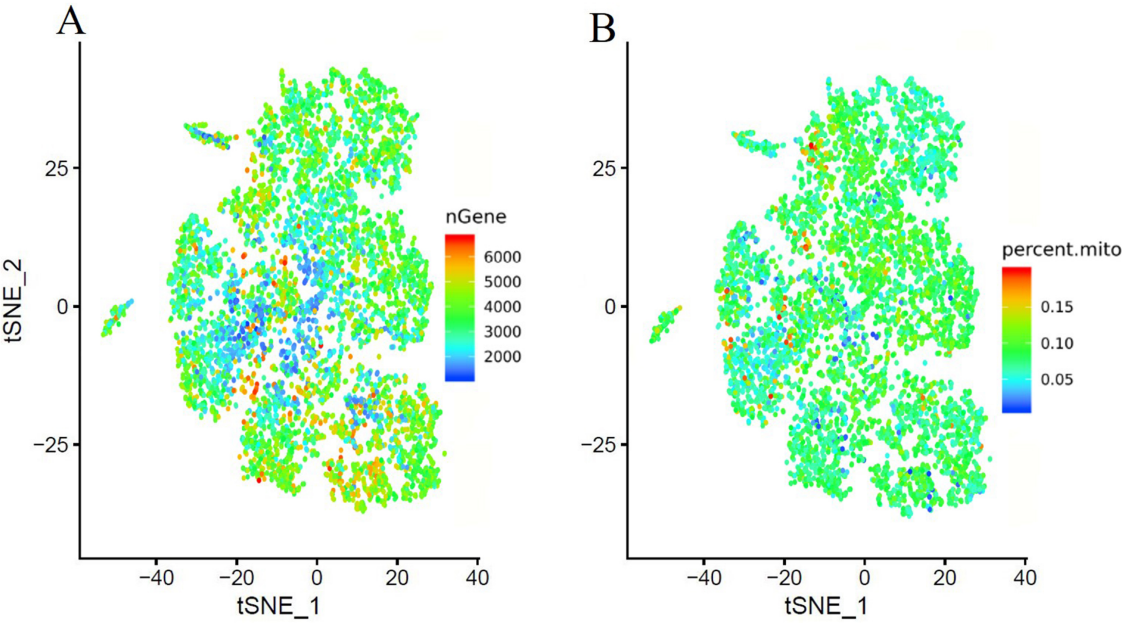

**Supplementary Figure S1. Cell quality for single cell sequencing.**

Supplement: Supplementary data [file annrheumdis-2019-215926supp001.pdf]

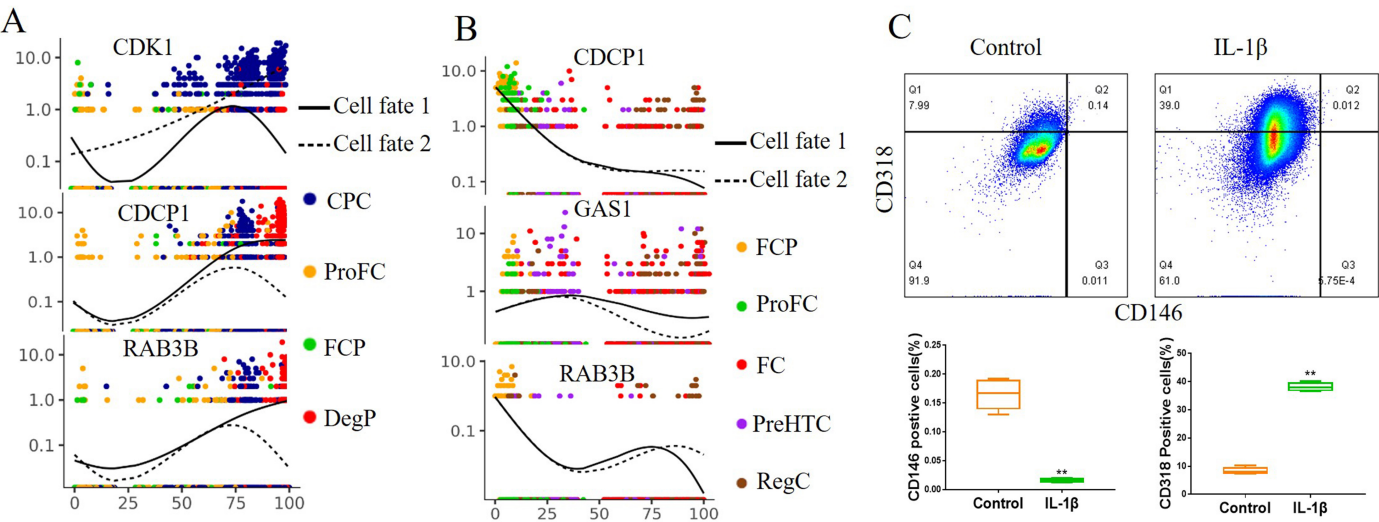

Supplementary Figure S5. Identification of DegP as a key element for meniscus degeneration.

Supplement: Supplementary data [file annrheumdis-2019-215926supp005.pdf]

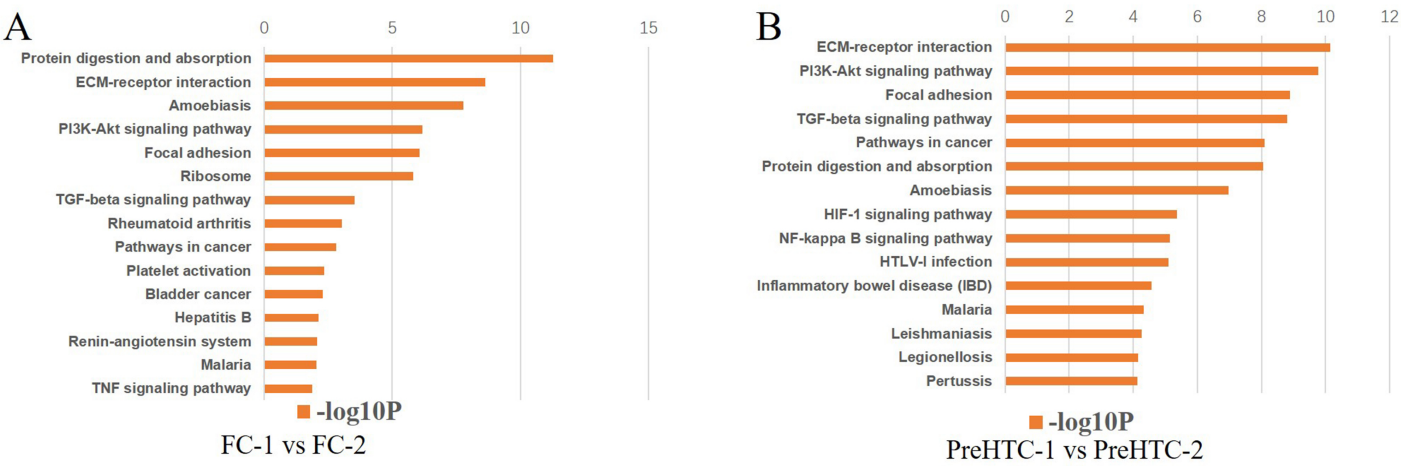

Supplementary Figure S6. TGFβ signalling pathway is upregulated in healthy human meniscus.

Supplement: Supplementary data [file annrheumdis-2019-215926supp006.pdf]
