# Supplementary material for: Single-cell RNA-seq analysis identifies meniscus progenitors and reveals the progression of meniscus degeneration
Source: Ann Rheum Dis. 2019 Dec 23;79(3):408–17. doi: 10.1136/annrheumdis-2019-215926 (PMC7034356; doi:10.1136/annrheumdis-2019-215926)
Supplement: Supplementary data [file annrheumdis-2019-215926supp002.pdf]

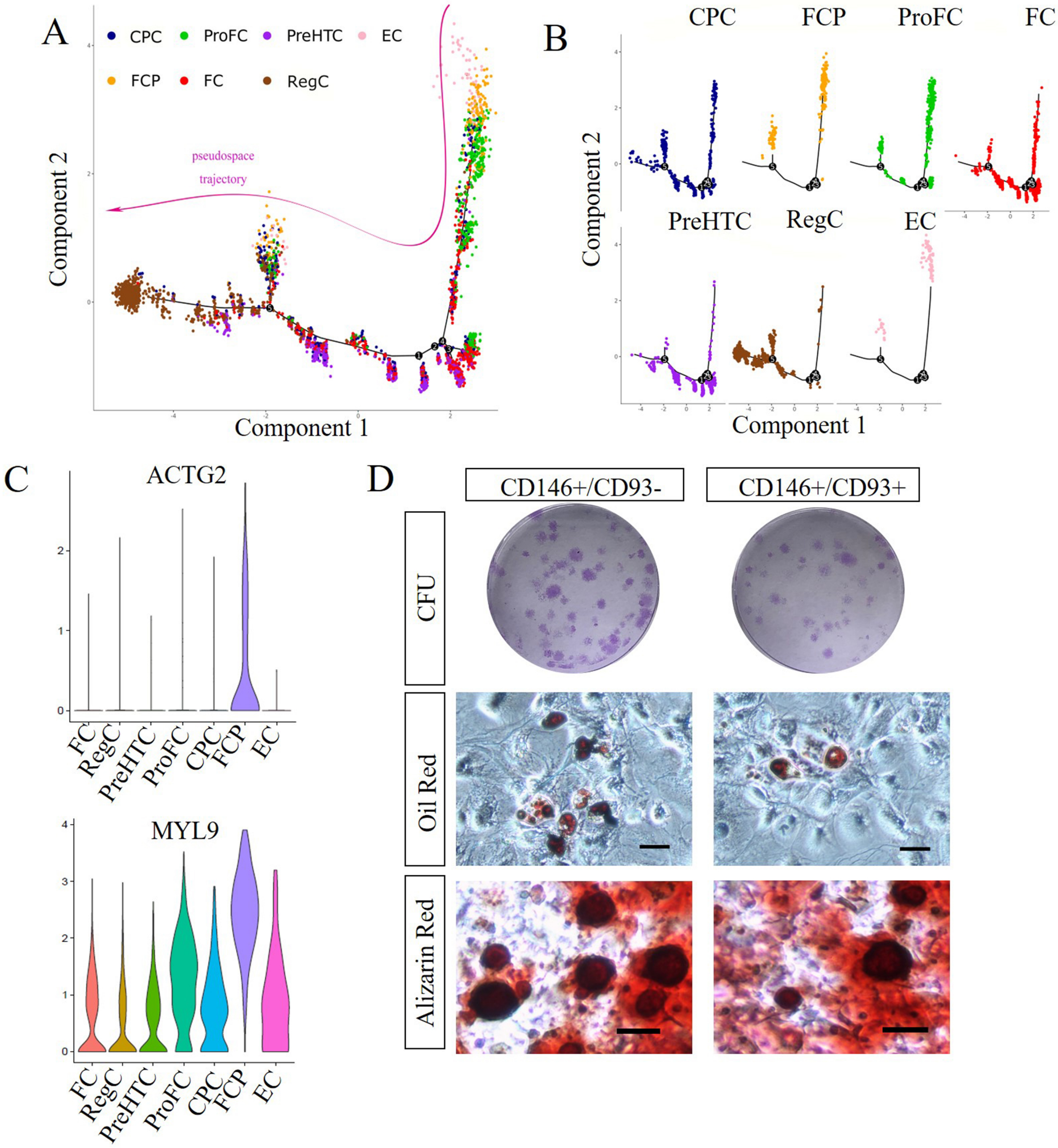

Supplementary Figure S2. Reconstruction of single-cell trajectory using Monocle 2 for healthy human meniscus cells.
