# Supplementary material for: Single-cell RNA-seq analysis identifies meniscus progenitors and reveals the progression of meniscus degeneration
Source: Ann Rheum Dis. 2019 Dec 23;79(3):408–17. doi: 10.1136/annrheumdis-2019-215926 (PMC7034356; doi:10.1136/annrheumdis-2019-215926)
Supplement: Supplementary data [file annrheumdis-2019-215926supp004.pdf]

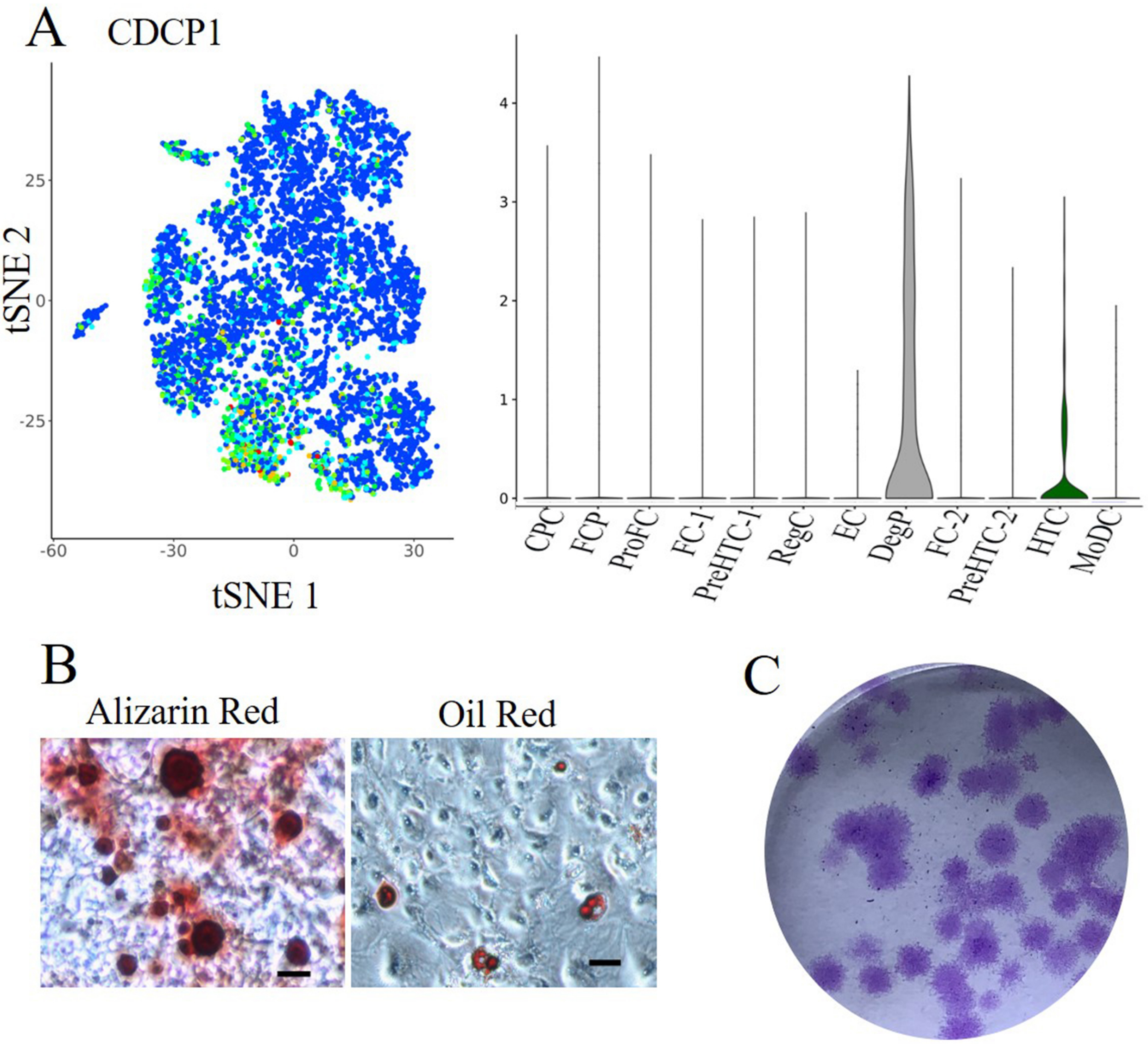

Supplementary Figure S4. CD318+ degenerated meniscus cells have characteristics of progenitor cells.
