# Supplementary material for: Single-cell RNA-seq analysis identifies meniscus progenitors and reveals the progression of meniscus degeneration
Source: Ann Rheum Dis. 2019 Dec 23;79(3):408–17. doi: 10.1136/annrheumdis-2019-215926 (PMC7034356; doi:10.1136/annrheumdis-2019-215926)
Supplement: Supplementary data [file annrheumdis-2019-215926supp007.pdf]

Supplementary Information

METHODS

Human meniscus samples

Human healthy meniscus samples were obtained from three male patients (mean ± SD age, 29.5 ± 6.6 years) who had no history of OA or rheumatoid arthritis and who underwent supra-knee amputation because of trauma, while human degenerated meniscus samples were obtained from four male patients (mean ± SD age, 58.5 ± 2.8 years) with knee OA who were undergoing knee arthroplasty surgery. We evaluate the severity of meniscus degeneration using Paudi's method.[1] The histological assessment indicated that the meniscus tissues in the healthy group were all Grade 1 (normal meniscus), while the degenerated group had 2 Grade 3 (moderate degeneration) and 2 Grade 4 (severe degeneration).

Culture of human meniscus cells

Meniscus cells were cultured in DMEM/Nutrient Mixture F-12 (Gibco Life Technology, Grand Island, NY, USA) containing 5% fetal bovine serum (FBS; Gibco Life Technology), 100 IU/mL penicillin, and 100 μg/mL streptomycin (Gibco Life Technology). Once the primary cells adhered to the culture plates, these cells were immediately used for single cell sequencing.

Flow cytometry analyses and cell sorting

For flow cytometry analyses, the cells were incubated with CD146 (FITC, #361012; Biolegend), CD318 (APC, #324008, Biolegend), or CD93(#336119; Biolegend) for 1 hour. Analyses were performed using BD LSR Fortessa SORP. Cell sorting was performed using BD FACS Aria III.

Colony-forming and multi-differentiation potential assays

After cell sorting, the cells were seeded at a low density to form colonies on 6-well

plate (2000 cells/well). After 7 days culture, the colonies were stained with 0.1% crystal violet.

For multi-differentiation assays, meniscus cells at 70% to 80% confluence in 6-well plates were treated with osteogenic differentiation induction medium (#HUXMA-90021; Cyagen Biosciences) for 7 days, or adipogenic differentiation induction medium (#HUXMA-90031; Cyagen Biosciences) for 21 days. Alizarin Red staining and Oil Red staining were performed for osteogenic and adipogenic differentiation, respectively.[2]

Mice meniscus surgery

A 3 mm longitudinal incision over the distal patella to proximal tibial plateau was used for mice meniscus surgery. The anterior meniscus was severed using micro-scissors. Then, the stability of medial meniscus was probed to confirm whether the meniscus had been completely cut off. After 28 days of surgery, mice were sacrificed for histology.

RT-PCR analysis and immunohistochemistry

Total cellular RNA was isolated using miRNeasy Mini Kit (Qiagen, Venlo, Netherlands). Next, cDNA was synthesized using PrimeScript™ RT Master Mix (Takara, Shiga, Japan). Transcript levels were normalized to that of the housekeeping gene glyceraldehyde 3-phosphate dehydrogenase (GAPDH). The specific primers used for these analyses are listed in Supplementary Table 1.

Immunohistochemical analysis was carried out as described previously.[2] For antigen retrieve, sections in 0.1% EDTA were incubated with moderate heat in microwave for 10 minutes. For staining, sections were treated with 3% normal goat serum for 1 h and incubated with antibodies specific to MYLK (#21642-1-AP; Proteintech), BMP2 (#66383-1-Ig; Proteintech), CD93 (#37276; Signalway Antibody), COL1A1 (#ab3471; Abcam), ZIP8 (#20459-1-AP; Proteintech), CDK1(#19532-1-AP; Proteintech), COL3A1 (#22734-1-AP; Proteintech), GAS1(#17903-1-AP; Proteintech), DNER (#DF10181-50; Affinity), TGFβ1(#21898-1-AP; Proteintech),

Polarized light microscopy

We used polarized light microscopy to compare the collagen structure of human healthy meniscus and degenerated meniscus. The sections were imaged under a 10× objective, from which quantitative maps of the retardation and angle were calculated at a resolution of 0.25 μm/pixel. The brightness of the retardation image represents the averaged fibril organization, where well-organized collagen fibres have a high retardation value.

Single-cell RNA-seq experiment

The BD Rhapsody System was used to obtain the transcriptomic information of single cells. Single-cell capture was achieved by random distribution of a single-cell suspension across >200,000 microwells using a limited dilution approach. Beads with oligonucleotide barcodes were added to saturation to pair the beads with the cells in the microwells. Cell-lysis buffer was added to hybridize poly-adenylated RNA molecules to the beads. Beads were collected into a single tube for reverse transcription. Upon cDNA synthesis, each cDNA molecule was tagged on the 5′ end (that is, the 3′ end of a mRNA transcript) with a unique molecular identifier (UMI) and cell label indicating its cell of origin. Whole transcriptome libraries were prepared using the BD Rhapsody single-cell whole-transcriptome amplification workflow. In brief, second strand cDNA was synthesized, followed by the ligation of the WTA adaptor for universal amplification. Eighteen cycles of PCR were used to amplify the adaptor-ligated cDNA products. Sequencing libraries were prepared using random priming PCR for the whole-transcriptome amplification products to enrich the 3′ end of the transcripts linked with the cell label and UMI. Sequencing libraries were quantified using a High Sensitivity DNA chip (Agilent) on a Bioanalyzer 2200 and the Qubit High Sensitivity DNA assay (Thermo Fisher Scientific). The library for each sample was sequenced by HiSeq Xten (Illumina, San Diego, CA) on a 150 bp paired-end run.

Single-cell RNA statistical analysis

We applied fastp with default parameters to filter the adaptor sequence and remove the low quality reads to obtain clean data.[3] UMI-tools was applied for single cell transcriptome analysis to identify the cell barcode whitelist.[4] The UMI-based clean data was mapped to the human genome (Ensemble version 91) utilizing STAR[5] mapping with customized parameters from the UMI-tools standard pipeline to determine the UMIs counts of each sample. In order to minimize the sample batch, we applied down sample analysis to the samples sequenced according to the mean reads per cell of each sample and achieved a cell expression table with a sample barcode. Cells containing over 200 expressed genes and a mitochondria UMI rate below 20% passed the cell quality filtering and mitochondria genes were removed in the expression table but used for cell expression regression to avoid the effect of the cell status for clustering analysis and marker analysis of each cluster.

Seurat package version 2.3.4 (https://satijalab.org/seurat/) was used for cell normalization and regression based on the expression table according to the UMI counts of each sample and the percentage mitochondria rate to obtain the scaled data. PCA was constructed based on the scaled data with all highly variable genes. The top 8 principals were used for tSNE construction.

Utilizing the cluster method (healthy meniscus group: k-mean method and K = 7; healthy meniscus group and degenerated meniscus group: graph cluster method and resolution = 1), we acquired the cell cluster result based on the PCA of the top 8 principals and calculated the marker genes using the Find All Markers function with the Wilcoxon Rank-Sum Test under following criteria: Log FC>0.25; p<0.05; min.pct>0.1.

Pseudotime analysis

Cells in different states express different sets of genes, producing a dynamic repertoire of proteins and metabolites that perform their roles in metabolism. As cells move between states, they undergo a process of transcriptional reconfiguration, with some

genes being silenced and others newly activated. Single cell sequencing presents gene expression changes within each cell. Every cell must go through these gene expression changes as part of a dynamic biological process. The trajectory represents the overall gene expression changes, and each cell has its proper position in the trajectory. If there are multiple outcomes for the process, the trajectory has different branches corresponding to cellular decisions.

We applied Single-Cell Trajectories analysis using Monocle2 (http://cole-trapnell-lab.github.io/monocle-release) with DDR-Tree and default parameters. Before Monocle analysis, we selected the marker genes of the Seurat clustering result. The raw expression counts of the cell passed filtering. Based on the pseudo-time analysis, branch expression analysis modelling (BEAM Analysis) was applied for branch fate determined gene analysis.

SCENIC analysis

The SCENIC analysis was run on the cells that passed the filtering, using the 20-thousand motifs database for RcisTarget and GRNboost, as previously described.[6]

Pathway analysis

Pathway analysis was used to identify the significant pathway of the differential genes according to the KEGG database. We used Fisher's exact test to select the significant pathway, where the threshold of significance was defined by the P-value and FDR. The cases were selected when $p<0.05$.[7]

Statistical analysis

Statistical calculations were performed using SPSS 17.0. The results of qRT-PCR in this article are displayed as mean±SD. The results of IHC and Flow are presented in box plots. The student's t tests were applied to identify differences between the two groups in IHC and qRT-PCR. Results were considered statistically significant when $P < 0.05$.

REFERENCES

1. C Pauli, SP Grogan, S Patil*, et al.* Macroscopic and histopathologic analysis of human knee menisci in aging and osteoarthritis. *Osteoarthritis Cartilage* 2011;19:1132-1141.
2. H Sun, X Zhao, C Zhang*, et al.* MiR-455-3p inhibits the degenerate process of chondrogenic differentiation through modification of DNA methylation. *Cell Death Dis* 2018;9:537.
3. S Chen, Y Zhou, Y Chen*, et al.* fastp: an ultra-fast all-in-one FASTQ preprocessor. *Bioinformatics* 2018;34:i884-i890.
4. T Smith, A Heger , I Sudbery. UMI-tools: modeling sequencing errors in Unique Molecular Identifiers to improve quantification accuracy. *Genome Res* 2017;27:491-499.
5. A Dobin, CA Davis, F Schlesinger*, et al.* STAR: ultrafast universal RNA-seq aligner. *Bioinformatics* 2013;29:15-21.
6. S Aibar, CB Gonzalez-Blas, T Moerman*, et al.* SCENIC: single-cell regulatory network inference and clustering. *Nat Methods* 2017;14:1083-1086.
7. S Draghici, P Khatri, AL Tarca*, et al.* A systems biology approach for pathway level analysis. *Genome Res* 2007;17:1537-1545.
