# Supplementary material for: Single-cell RNA-seq analysis identifies meniscus progenitors and reveals the progression of meniscus degeneration
Source: Ann Rheum Dis. 2019 Dec 23;79(3):408–17. doi: 10.1136/annrheumdis-2019-215926 (PMC7034356; doi:10.1136/annrheumdis-2019-215926)
Supplement: Supplementary data [file annrheumdis-2019-215926supp008.pdf]

Supplementary Table 1: Primers for quantitative real-time polymerase chain reaction (qRT-PCR).

Gene		Primer sequence (5'-3')
hsa-COL1A1	F	GAGGGCCAAGACGAAGACATC
hsa-COL1A1	R	CAGATCACGTCATCGCACAAC
hsa-COL3A1	F	GCCAAATATGTGTCTGTGACTCA
hsa-COL3A1	R	GGGCGAGTAGGAGCAGTTG
hsa-MMP1	F	CTCTGGAGTAATGTCACACCTCT
hsa-MMP1	R	TGTTGGTCCACCTTTCATCTTC
hsa-MMP3	F	CTGGACTCCGACACTCTGGA
hsa-MMP3	R	CAGGAAAGGTTCTGAAGTGACC
hsa-CDK1	F	AAACTACAGGTCAAGTGGTAGCC
hsa-CDK1	R	TCCTGCATAAGCACATCCTGA
hsa-CDCP1	F	CTGAACTGCGGGGTCTCTATC
hsa-CDCP1	R	GTCCCCAGCTTTATGAGAACTG
hsa-DNER	F	AAGGCTATGAAGGTCCCAACT
hsa-DNER	R	CTGAGAGCGAGGCAGGATTT
hsa-MCAM	F	AGCTCCGCGTCTACAAAGC
hsa-MCAM	R	CTACACAGGTAGCGACCTCC
hsa-S100A9	F	GGTCATAGAACACATCATGGAGG
hsa-S100A9	R	GGCCTGGCTTATGGTGGTG
hsa-CD93	F	CCGGAAGTAACATTGAGGGCT
hsa-CD93	R	TCTGAGTCTCGTCCTTGTCAC
hsa-GAPDH	F	GCACCGTCAAGGCTGAGAAC
hsa-GAPDH	R	TGGTGAAGACGCCAGTGGA
